# Supplementary material for: Papillary-Muscle-Derived Radiomic Features for Hypertrophic Cardiomyopathy versus Hypertensive Heart Disease Classification
Source: Diagnostics (Basel). 2023 Apr 25;13(9):1544. doi: 10.3390/diagnostics13091544 (PMC10177511; doi:10.3390/diagnostics13091544)
Supplement: Supplementary file 1 [file diagnostics-13-01544-s001.zip › diagnostics-2344859-supplementary.pdf]

Myocardium and papillary muscle derived radiomic features for left ventricular hypertrophy detection and hypertrophic cardiomyopathy versus hypertensive heart disease classification

Supplementary Materials

Supplementary methods	Page 2
Supplementary figures	Pages 4-5
Supplementary tables	Pages 6-12
Reference	Page 13

Supplementary methods

Image processing

Image processing and feature extraction were performed with Pyradiomics library (version 3.0.1) in a Python environment (version 3.7.10) in compliance with the latest image biomarker standardisation initiative (IBSI) documentation [1,2].

In this study, image processing included following steps: 1) resampling: images were resampled to 1.0×1.0×1.0 mm using appropriate interpolation method (B-spline interpolation for images and nearest neighbor for masks); 2) grey level normalization and discretization: VOIs were normalized and discretized to a fixed bin width of 16.

Supplementary figures

Supplementary figure S1. An example of apical basal muscle bundle.

An example of apical-basal muscle bundle that is hard to distinguish from papillary muscle at short-axis cine images in a 59 years old male HCM patient. **(a)** Long axis 2 chamber view at end-diastole of this patient, apical-basal muscle bundle was denoted with white arrow. **(b)** short axis view at the end diastole, from top left (basal) to down right (apical), the apical-basal muscle bundle was denoted with a white triangle, in the short axis view, this muscle bundle is easily confused with papillary muscle.

Supplementary figure S2. Comparison of models using calibration curves.

Calibtration curves for different models were plotted, (a) showed calibration curves for detection task with MYO group; (b) showed calibration curves for differentiation task with MYO+PM group.

Supplementary Figure S1.

(a) Long axis 2 chamber at end diastole (b) Short axis at end diastole from base to apex

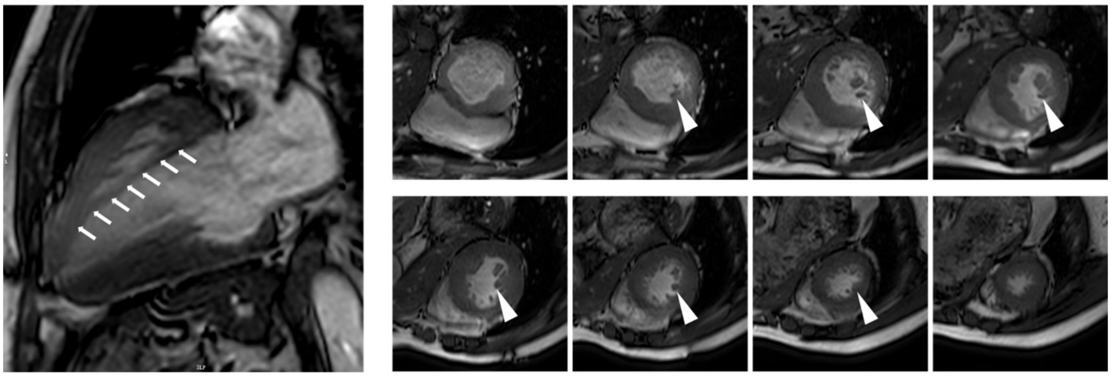

Supplementary Figure S2.

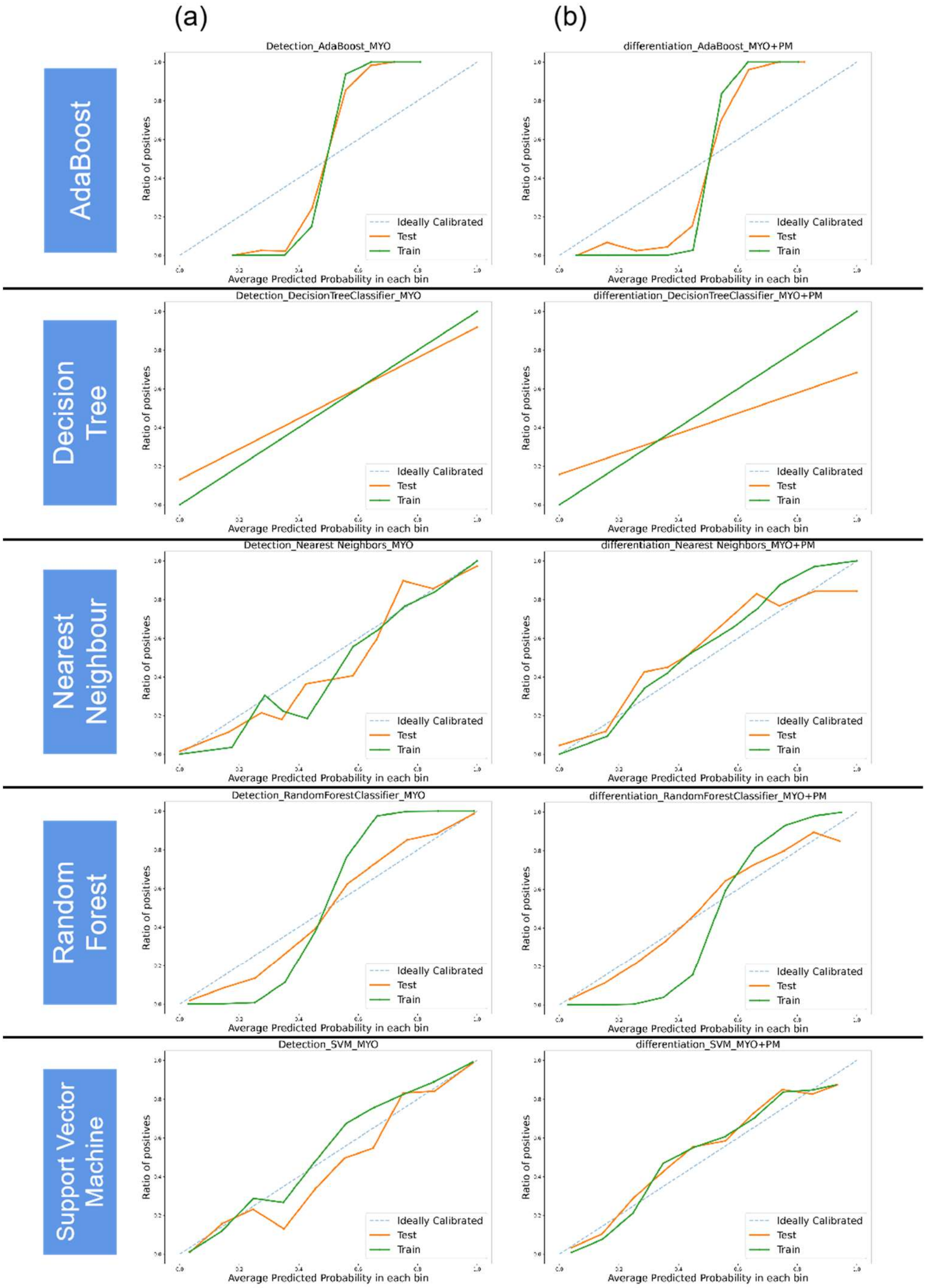

Supplementary Table S1. Full list of extracted features.

VOI	Filter	Type		Number	Total
MYO	Original	Shape		14	1316
	Original: 1<br>LOG: 4<br>Wavelet: 8<br>Gradient: 1<br>Total: 14	Firstorder		18×14 (Total Filters)	
		Texture	NGTDM	5×14 (Total Filters)	
			GLCM	24×14 (Total Filters)	
			GLDM	14×14 (Total Filters)	
			GLRLM	16×14 (Total Filters)	
			GLSZM	16×14 (Total Filters)	
PM	Original	Shape		14	1316
	Original: 1<br>LOG: 4<br>Wavelet: 8<br>Gradient: 1<br>Total: 14	Firstorder		18×14 (Total Filters)	
		Texture	NGTDM	5×14 (Total Filters)	
			GLCM	24×14 (Total Filters)	
			GLDM	14×14 (Total Filters)	
			GLRLM	16×14 (Total Filters)	
			GLSZM	16×14 (Total Filters)	

GLCM: gray level co-occurrence matrix; GLDM: gray level dependence matrix; GLRLM : gray level run length matrix; GLSZM: gray level size zone matrix; LOG: Laplace of Gaussian; NGTDM: Neighboring gray tone difference matrix.

Supplementary Table S2. Full list of features ranked using Boruta method for detection task.

	MYO (N = 28)	Importance[#]	PM (N = 32)	Importance[#]
1	wavelet-LHL_glcm_Correlation	54.9	original_shape_Maximum2DDiameterRow	1.1
2	original_shape_Sphericity	11.0	wavelet-HLH_glcm_MCC	0.9
3	wavelet-HHH_firstorder_Median	9.8	wavelet-HHH_firstorder_Uniformity	0.8
4	wavelet-LHH_firstorder_Energy	4.4	wavelet-HHH_glcm_SumEntropy	0.4
5	wavelet-LHH_firstorder_Kurtosis	1.9	wavelet-LHH_glcm_ClusterShade	0.4
6	log-sigma-2-0-mm-3D_glcm_ClusterShade	1.5	wavelet-LLH_ngtdm_Contrast	0.3
7	wavelet-LLH_glrlm_RunEntropy	1.3	wavelet-HLL_glcm_Correlation	0.3
8	wavelet-LHL_glcm_Imc2	0.8	original_shape_Flatness	0.3
9	wavelet-HLL_glcm_InverseVariance	0.6	wavelet-LHL_glcm_Imc1	0.2
10	wavelet-HLL_firstorder_Maximum	0.4	wavelet-LHH_ngtdm_Strength	0.2
1	wavelet-HHL_ngtdm_Contrast	0.3	wavelet-HLH_firstorder_Uniformity	0.1

1				
12	log-sigma-2-0-mm-3D_glcm_Correlation	0.3	gradient_firstorder_Minimum	0.1
13	gradient_glcm_Correlation	0.2	wavelet-LHL_firstorder_Kurtosis	0.1
14	wavelet-HLL_glcm_Correlation	0.2	wavelet-HHH_glszm_GrayLevelNonUniformityNormalized	0.1
15	wavelet-LLH_ngtdm_Strength	0.2	original_shape_Elongation	0.1
16	wavelet-LHH_glcm_ClusterShade	0.2	wavelet-HHL_firstorder_Kurtosis	0.1
17	wavelet-LLH_glcm_Contrast	0.2	wavelet-HHH_glszm_GrayLevelNonUniformity	0.1
18	log-sigma-2-0-mm-3D_gldm_LargeDependenceHighGrayLevelEmphasis	0.2	wavelet-HLH_firstorder_Kurtosis	0.1
1	original_shape_Flatness	0.2	wavelet-HHL_gldm_DependenceNonUniformityN	<0.1

9			ormalized	
20	wavelet-LHH_glszm_GrayLevelNonUniformity	<0.1	wavelet-HLH_firstorder_Mean	<0.1
21	wavelet-HHL_glcm_MCC	<0.1	wavelet-HHL_ngtdm_Contrast	<0.1
22	wavelet-LLH_firstorder_Mean	<0.1	wavelet-LHL_glszm_ZoneEntropy	<0.1
23	wavelet-HHL_glcm_InverseVariance	<0.1	wavelet-HHL_glcm_ClusterShade	<0.1
24	wavelet-LHL_glcm_ClusterShade	<0.1	wavelet-LLH_glszm_LargeAreaLowGrayLevelEmphasis	<0.1
25	wavelet-HLH_firstorder_Minimum	<0.1	wavelet-LHL_gldm_SmallDependenceLowGrayLevelEmphasis	<0.1
26	wavelet-HLL_glcm_Imc2	<0.1	wavelet-LHH_gldm_DependenceNonUniformityNormalized	<0.1
2	original_firstorder_Maximum	<0.1	wavelet-LLH_glcm_Correlation	<0.1

7				
28	wavelet-HHH_ngtdm_Strength	<0.1	wavelet-HHH_glszm_SizeZoneNonUniformityNormalized	<0.1
29			wavelet-HHL_glcm_Correlation	<0.1
30			wavelet-LHL_firstorder_Skewness	<0.1
31			wavelet-HHH_firstorder_Maximum	<0.1
32			wavelet-LLH_glcm_Idmn	<0.1

[#] The maximal shadow feature importance is 0.2.

GLCM: gray level co-occurrence matrix; GLDM: gray level dependence matrix; GLRLM : gray level run length matrix; GLSZM: gray level size zone matrix; LOG: Laplace of Gaussian; NGTDM: Neighboring gray tone difference matrix.

Other abbreviation for specific features could be found in Pyradiomics documentation.

Supplementary Table S3. Full list of features ranked using Boruta method for differentiation task.

	MYO (N = 8)	Importance#	PM (N = 12)	Importance#
1	gradient_glcm_Correlation	37.5	original_shape_Maximum2DDiameterSlice	10.4
2	original_shape_Sphericity	4.9	log-sigma-5-0-mm-3D_firstorder_Kurtosis	3.6
3	original_shape_Elongation	3.5	original_glszm_ZoneEntropy	3.2
4	wavelet-LHL_glcm_Imc1	3.0	wavelet-HLL_glcm_Imc2	3.0
5	log-sigma-5-0-mm-3D_glszm_ZoneEntropy	1.3	log-sigma-2-0-mm-3D_glcm_Correlation	2.9
6	wavelet-LHH_glcm_MCC	1.2	gradient_glcm_Idmn	2.4
7	log-sigma-2-0-mm-3D_firstorder_10Percentile	1.0	wavelet-HHL_glcm_Correlation	2.0
8	wavelet-LHH_glszm_GrayLevelNonUniformityNormalized	0.7	log-sigma-3-0-mm-3D_glcm_Imc1	1.3
9			wavelet-LLH_glcm_MCC	0.8
10			wavelet-HLH_glcm_MCC	0.7
11			gradient_firstorder_Kurtosis	0.7
12			original_firstorder_Kurtosis	0.5

[#] The maximal shadow feature importance is 0.9.

GLCM: gray level co-occurrence matrix; GLDM: gray level dependence matrix; GLRLM : gray level run length matrix; GLSZM: gray level size zone matrix; LOG: Laplace of Gaussian; NGTDM: Neighboring gray tone difference matrix.

Other abbreviation for specific features could be found in Pyradiomics documentation.

Reference:

[1] A. Zwanenburg, M. Vallières, M.A. Abdalah, H.J.W.L. Aerts, V. Andrearczyk, A. Apte, S. Ashrafinia, S. Bakas, R.J. Beukinga, R. Boellaard, M. Bogowicz, L. Boldrini, I. Buvat, G.J.R. Cook, C. Davatzikos, A. Depeursinge, M.-C. Desseroit, N. Dinapoli, C.V. Dinh, S. Echegaray, I. El Naqa, A.Y. Fedorov, R. Gatta, R.J. Gillies, V. Goh, M. Götz, M. Guckenberger, S.M. Ha, M. Hatt, F. Isensee, P. Lambin, S. Leger, R.T.H. Leijenaar, J. Lenkowicz, F. Lippert, A. Losnegård, K.H. Maier-Hein, O. Morin, H. Müller, S. Napel, C. Nioche, F. Orlhac, S. Pati, E.A.G. Pfaehler, A. Rahmim, A.U.K. Rao, J. Scherer, M.M. Siddique, N.M. Sijtsema, J. Socarras Fernandez, E. Spezi, R.J.H.M. Steenbakkers, S. Tanadini-Lang, D. Thorwarth, E.G.C. Troost, T. Upadhaya, V. Valentini, L.V. van Dijk, J. van Griethuysen, F.H.P. van Velden, P. Whybra, C. Richter, S. Löck, The Image Biomarker Standardization Initiative: Standardized Quantitative Radiomics for High-Throughput Image-based Phenotyping, Radiology. 295 (2020) 328–338. https://doi.org/10.1148/radiol.2020191145.

[2] J.J.M. van Griethuysen, A. Fedorov, C. Parmar, A. Hosny, N. Aucoin, V. Narayan, R.G.H. Beets-Tan, J.-C. Fillion-Robin, S. Pieper, H.J.W.L. Aerts, Computational Radiomics System to Decode the Radiographic Phenotype, Cancer Res. 77 (2017) e104–e107. https://doi.org/10.1158/0008-5472.CAN-17-0339.
